# Clinicoradiographic Features and Histopathologic Variations of Intraosseous Lipoma: Report of a Case and Review of the Literature

**DOI:** 10.1155/2021/2073001

**Published:** 2021-07-03

**Authors:** Saede Atarbashi-Moghadam, Ali Lotfi, Mohammad Mehdizadeh, Fazele Atarbashi-Moghadam

**Affiliations:** ^1^Department of Oral and Maxillofacial Pathology, School of Dentistry, Shahid Beheshti University of Medical Sciences, Tehran, Iran; ^2^Department of Oral and Maxillofacial Surgery, School of Dentistry, Qom University of Medical Sciences, Qom, Iran; ^3^Department of Periodontics, School of Dentistry, Shahid Beheshti University of Medical Sciences, Tehran, Iran

## Abstract

Intraosseous lipoma is a very rare lesion, and the jaw is its least common bone location. Fibrolipoma is a microscopic variant of lipoma which is characterized by a significant fibrous component intermixed with lobules of fat cells. Intraosseous fibrolipoma of the jaw is a rare lesion, and to the best of our knowledge, only two cases have been reported from 1948 in English literature. This paper presents a 39-year-old man with a chief complaint of tooth displacement in the anterior region of the mandible. Radiographic evaluation revealed a unilocular radiolucent lesion with sclerotic borders located between the left lateral incisor and canine. Histopathologic evaluation after an excisional biopsy confirmed the diagnosis of intraosseous fibrolipoma. We also reviewed the literature on this rare lesion.

## 1. Introduction

Intraosseous lipoma (IOL) is the rarest benign primary bone neoplasm and accounts for about 0.1% of all bone tumors. Its commonest location is the metaphysis of the long bones and the calcaneus [1]. It is often discovered incidentally during radiographic examinations, although it may be associated with pathological fractures. IOLs are almost always solitary but multiple lesions have also been reported [2]. IOLs of long bones show discrete osteolytic lesions, often with sclerotic border and radioopacities in the center due to fatty necrosis and calcification [1]. There is no sex predilection in IOLs of long bone [2].

There are few reports of IOL in the jaw, and the cause of this lesion is not known [1, 3–8]. It is usually asymptomatic; although pain, swelling, and paraesthesia have been reported [1, 4]. It is found incidentally on radiographs that show a well-defined radiolucency [4]. This lesion has clinical similarities with several other radiolucent jaw lesions, and its definitive diagnosis needs histopathologic examination [4, 5]. Recurrence or malignant transformation of IOL in the maxillofacial region has not been reported in the literature. Fibrolipoma is a variant of conventional lipoma composed of mature fat cells intermixed with fibrous connective tissue. Conservative surgery is the treatment of choice [3]. This paper presents a case of intraosseous fibrolipoma of the mandible afflicting a 39-year-old man and reviews the literature on this rare lesion.

## 2. Case Report

**Figure 1:**
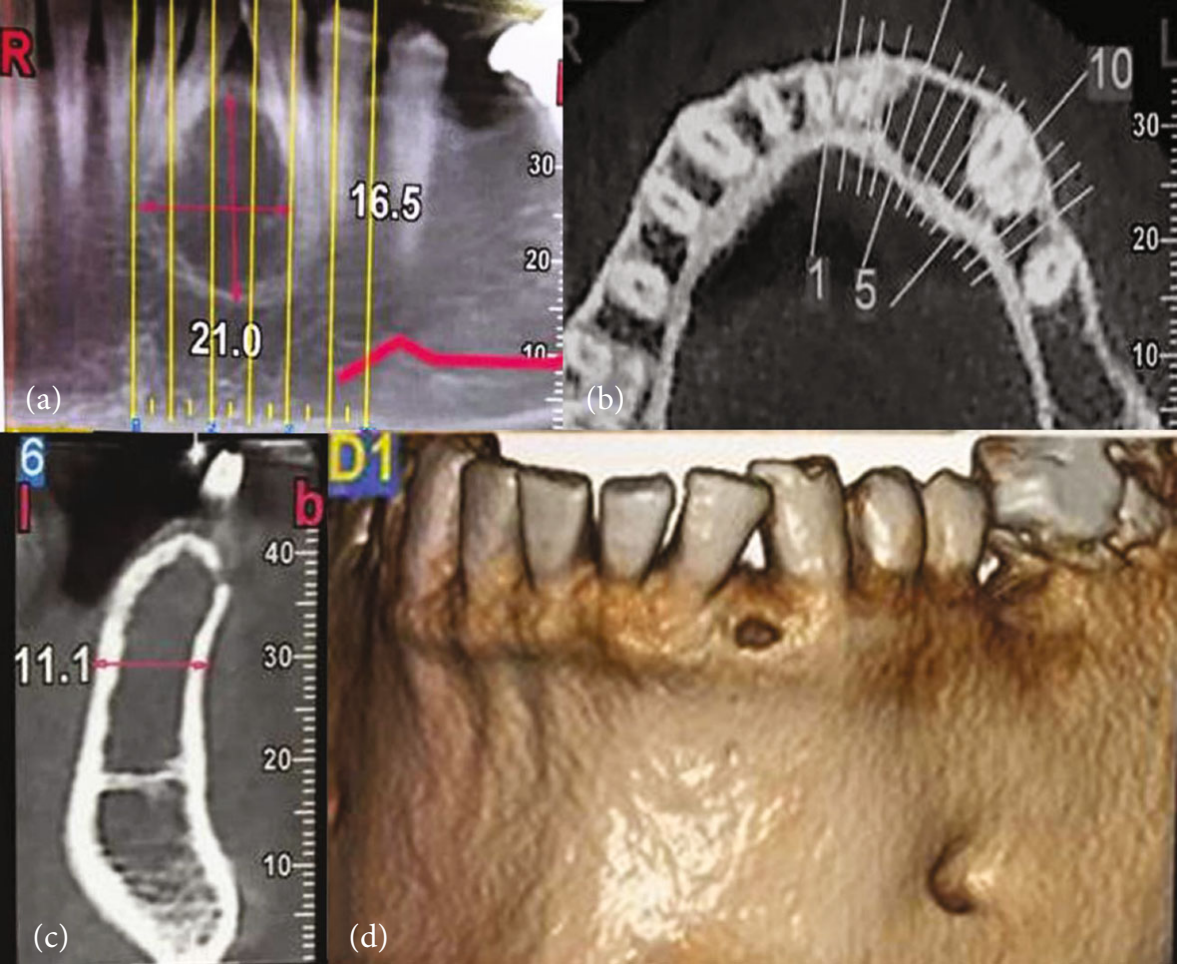
Cone beam computed tomography assessment of the lesion: (a) a well-defined radiolucent lesion with root separation in panoramic view; (b) mesiodistal extension of the lesion in axial view; (c) cross-sectional view; (d) 3D assessment.

A 39-year-old man with a chief complaint of tooth displacement in the mandibular left lateral incisor and canine area was referred to a private dental clinic (Tehran, Iran) in November 2019. There was neither a history of medical problems nor trauma. Extraoral examination was within normal limits and the intraoral examination revealed no expansion or inflammation. The displaced teeth were vital. Cone-beam computed tomography (CBCT) revealed an approximately 2 cm, unilocular radiolucent lesion with sclerotic borders located between the mandibular left lateral incisor and canine which causes root divergence of the involved teeth (Figure 1). There was no evidence of root resorption.

**Figure 2:**
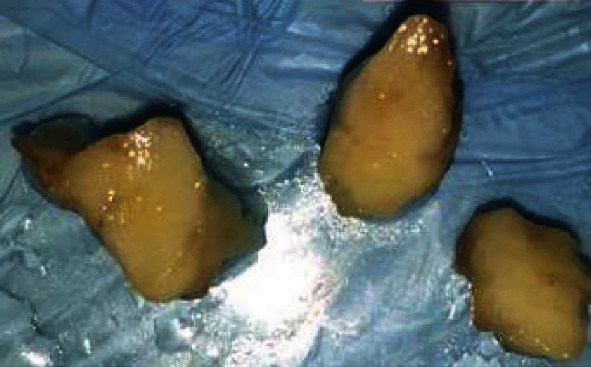
Gross of the lesion with solid and yellow cut surface.

**Figure 3:**
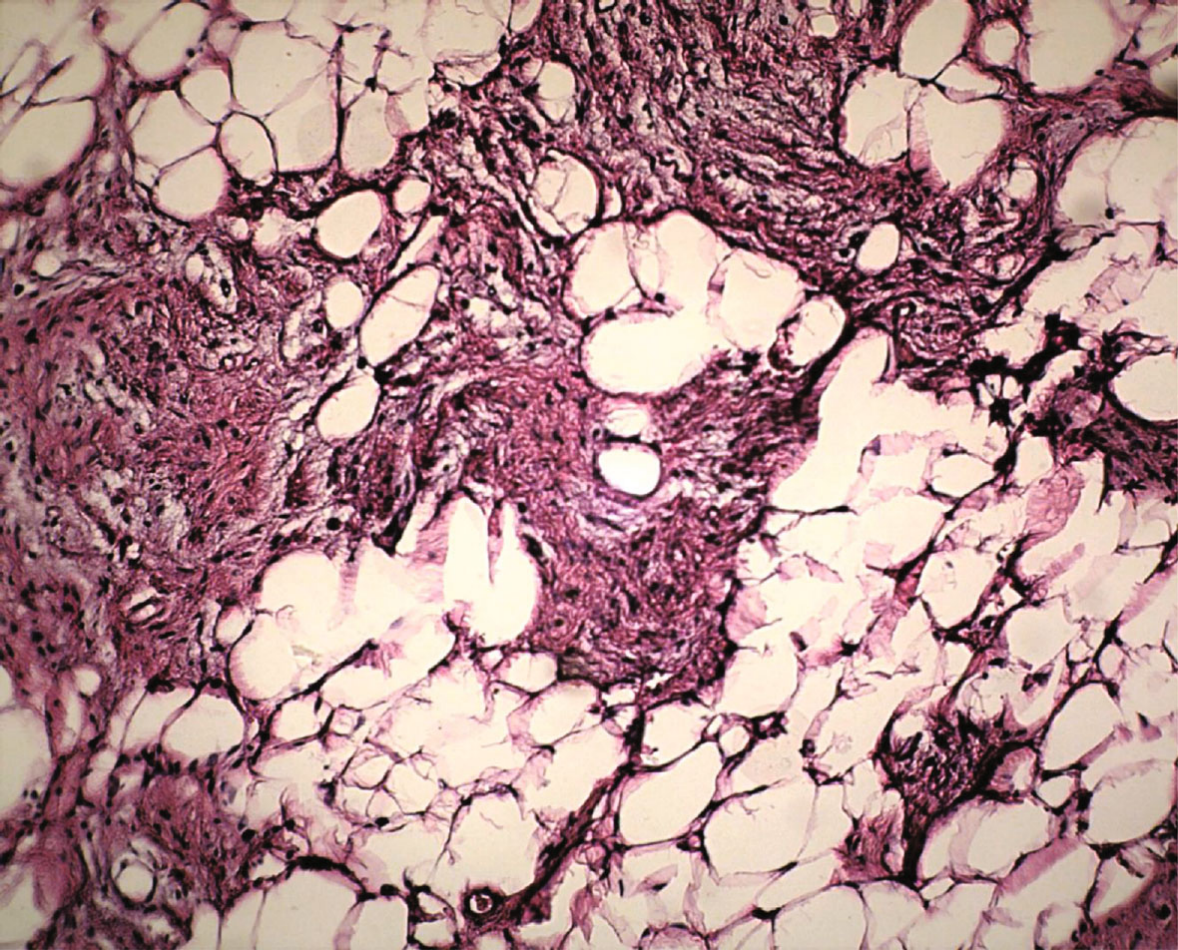
The proliferation of fatty tissue intermixed with fibrous connective tissue (H&E, 100x).

Lateral periodontal cyst, odontogenic keratocyst, and central giant cell granuloma were considered as differential diagnoses. To make a final diagnosis, an excisional biopsy was performed under local anesthesia in the same month. The gross of the specimen showed a well-defined, solid, yellow lesion with soft consistency (Figure 2). Microscopic examination showed a well-defined mass composed of mature fat cells intermixed with fibrous connective tissue (Figure 3). The histopathological features in combination with radiologic findings were consistent with the diagnosis of intraosseous fibrolipoma. The patient has been under follow-up and free of disease until now.

## 3. Discussion

**Figure 4:**
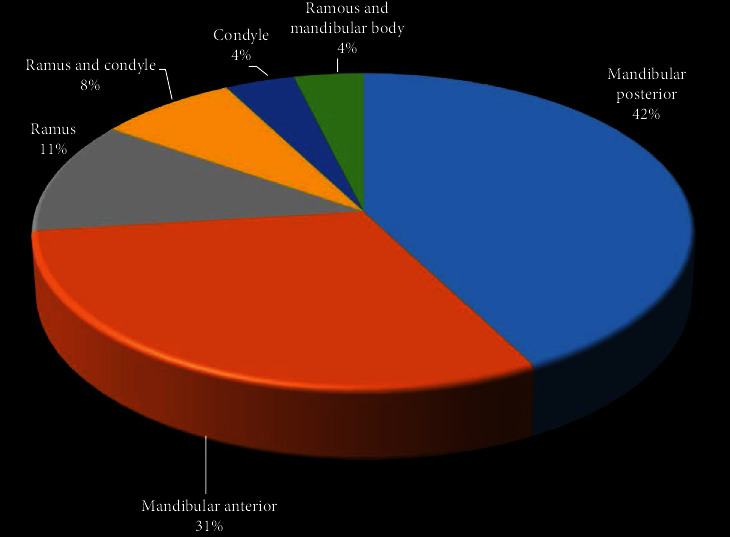
Frequency of intraosseous lipoma in different regions of the mandible in patients reported with intraosseous lipoma described in the literature and present case.

Although lipocytes are present throughout the bone marrow, intraosseous type of lipoma rarely occurs [6]. IOL is divided into intramedullary and intracortical groups [4]. Our literature review of IOL in the PubMed database (with sufficient information) from 1948 to November 2020 resulted in 29 cases. The demographic, clinical, and radiographic features of these cases and the present case are summarized and presented in Table 1. According to this review, the female to male ratio was equal. However, previous studies stated a male predilection or equal tendency for both sexes [6, 7]. IOL of the jaws, like long bone IOL, can develop in almost any age group with a mean age of 42.66 ± 14.28 (ranging from 15 to 65 years) but occurs most often in the 6^th^ decade of life. The mandible was more afflicted than the maxilla (86.66%). All maxillary cases were in the posterior region; moreover, there is a posterior predilection for mandibular lesions (Figure 4). About 46.66% of IOLs were asymptomatic, although swelling, pain, chin numbness, hypoesthesia, trismus, and periodontal signs are also reported [9–13]. Burić et al. [6] mentioned that the symptoms of the IOL are mostly related to the location and size of the lesion. The present case showed root divergence of adjacent teeth without signs of resorption (Figure 1). Almost all cases were intramedullary (93.33%), while one periosteal case and one parosteal case were reported [11, 14]. The majority of lesions were radiolucent, though 6.66% of cases showed mixed radiolucent/radiopaque radiography [4, 15]. IOL usually shows uncharacteristic radiography and demonstrates a cyst-like lesion with increased radiolucency surrounded by a sclerotic rim [3]. A multilocular lesion was also reported [13]. Therefore, microscopic examination is mandatory for diagnosis [6].

Histopathologic examination of IOL shows mature adipocytes without atypia and hematopoietic tissue that may be encapsulated [8]. Fibrolipoma is a variant of conventional lipoma composed of mature fat cells intermixed with fibrous connective tissue [3]. In the current review, the most common subtype was angiolipoma (16.66%), followed by fibrolipoma (10%) and spindle cell lipoma (3.33%). Spindle cell lipoma is also rare among all soft tissue lipomas and accounts for about 1.5% of cases [9]. The etiology of IOL is not clear; however, the role of factors such as trauma, infarction, inflammation, and nutritional problems has been suggested [1, 3, 6]. In the presented case, there was not any history of trauma or tooth extraction in this region. In addition, Burić et al. [6] considered the chronic bacterial infection of retained roots as a causative factor by creating a bone defect in the periapical region, which allows the easy accumulation of fat cells and the formation of a lipoma in the bone. In our case, the adjacent teeth were vital without any symptoms of pulpitis or periapical infection. Moreover, some of the IOLs were found in the ramus area without any relationship to the teeth [3, 8]. In the study of Barker and Sloan [7], patients were over 50 years old; therefore, they suggested that these findings represented fatty degeneration of the bone marrow. However, in the present literature review, about 43.33% of patients were under 40 years old; thus, fatty degeneration of bone marrow can be ruled out. Conservative surgery is the treatment of choice, and recurrence or malignant transformation of IOL in the maxillofacial region has not been reported in the literature [3]. However, malignant transformation of IOL in other sites has been reported [16].

## 4. Conclusion

As IOL is infrequent, each new case must be documented. To the best of our knowledge, only three cases of intraosseous fibrolipoma of the mandible (including our case) have been reported since 1948. IOLs of the jaws affect both genders equally, and it occurs most often in the 6th decade of life. The majority of cases are asymptomatic, although swelling, pain, paresthesia, and root divergence may be encountered.

## Figures and Tables

**Table 1:** Demographic, clinical, and radiographic data of patients reported with intraosseous lipoma described in the literature and present case.

Variables	
Patients (*n*)	30
Male (%)/female (%)	15 (50)/15 (50)
Age (years), mean ± SD (min-max)	42.66 ± 14.28 (15-65)
Position (%)	
Maxilla	4 (13.33)
Mandible	26 (86.66)
Clinical feature	
Asymptomatic (%)	14 (46.66)
Symptomatic (%)	16 (53.33)
Radiographic feature	
Radiolucent	27 (90)
Mixed	3 (10)
Pathological diagnosis	
Lipoma	21 (70)
Angiolipoma	5 (16.66)
Fibrolipoma	3 (10)
Spindle cell lipoma	1 (3.33)

## Data Availability

The data supporting the findings of this study are available within the article and its supplementary materials.

## Supplementary Materials

Supplementary Materials Details of PubMed published cases of intraosseous lipoma of jaws from 1948 to Nov 2020 and present case.
