# Supplementary material for: Clinicoradiographic Features and Histopathologic Variations of Intraosseous Lipoma: Report of a Case and Review of the Literature
Source: Case Rep Dent. 2021 Jul 3;2021:2073001. doi: 10.1155/2021/2073001 (PMC8275430; doi:10.1155/2021/2073001)
Supplement: Supplementary Materials — Details of PubMed published cases of intraosseous lipoma of jaws from 1948 to Nov 2020 and present case. [file 2073001.f1.docx]

Supplementary file: Details of PubMed published cases of intraosseous lipoma of jaws from 1948 to Nov 2020 and present case

| **No** | **Author** | **Age** | **Sex** | **Jaws** | **Location** | **Symptoms** | **Radiographic finding** | **Histopathologic diagnosis** |
| --- | --- | --- | --- | --- | --- | --- | --- | --- |
| 1 | Tomo et al 2020 | 62 | M | Mandible | posterior | asymptomatic | Radiolucent | Angiolipoma |
| 2 | Dehghani et al 2019 | 33 | F | Mandible | Anterior | asymptomatic | Radiolucent | Lipoma |
| 3 | Tabakovic et al 2019 | 43 | F | Maxilla | posterior | periodic pains & stupor | Radiolucent | Lipoma |
| 4 | Babu et al 2019 | 52 | M | Maxilla | posterior | painful swelling | Radiolucent | Lipoma |
| 5 | Shin et al 2017 | 39 | F | Mandible | Condyle | Mouth opening limitation* | Radiolucent | Lipoma |
| 6 | Waśkowska et al 2017 | 32 | M | Mandible | Anterior | Asymptomatic | Radiolucent | Lipoma |
| 7 | Cooper et al 2017 | 53 | M | Mandible | posterior | swelling | Radiolucent | Spindle cell lipoma |
| 8 | Sanjuan et al 2015 | 50 | F | Mandible | Ramus & condyle | Asymptomatic | Radiolucent | Lipoma |
| 9 | Castellani et al 2015 | 25 | F | Mandible | Ramus | Asymptomatic | Radiolucent | Fibrolipoma |
| 10 | Basheer et al 2013 | 15 | M | Mandible | Anterior | Swelling | Hazy radiopacitis | Lipoma |
| 11 | Sun et al 2013 | 48 | M | Mandible | Anterior | Swelling & chin numbness | Irregular area of ossification | Lipoma (Ossifying Parosteal) |
| 12 | Hemavathy et al 2012 | 21 | F | Mandible | Ramus and body | Swelling | Radiolucent | Angiolipoma |
| 13 | Morais et al 2011 | 39 | F | Maxilla | posterior | Asymptomatic | Radiolucent | Lipoma |
| 14 | González-Pérez et al 2010 | 61 | F | Mandible | Ramus and condyle | Pain, trismus | Radiolucent | Lipoma |
| 15 | Cakarer et al 2009 | 45 | F | Mandible | Anterior | Asymptomatic | Radiolucent | Lipoma |
| 16 | Darling et al 2005 | 22 | F | Mandible | Anterior | Asymptomatic | Radiolucent | Lipoma |
| 17 | Keogh et al 2004 | 56 | F | Mandible | Posterior | Asymptomatic | Radiolucent | Lipoma |
| 18 | Buric et al 2001 | 62 | F | Mandible | Anterior | Swelling | Radiolucent | Lipoma |
| 19 | Sakashita et al 1998 | 17 | M | Maxilla | Posterior | Asymptomatic | Radiolucent | Lipoma |
| 20 | Manganaro et al 1994 | 51 | M | Mandible | Ramus | Asymptomatic | mixed | Angiolipoma |
| 21 | Barker and Sloan 1986 | 53 | F | Mandible | Posterior | Asymptomatic | Radiolucent | Lipoma |
| 22 | Heir and Geron 1983 | 43 | F | Mandible | Ramus | Hypoesthesia | Radiolucent | Lipoma |
| 23 | Miller et al 1982 | 51 | M | Mandible | Posterior | Periodontal symptoms | Radiolucent | Lipoma |
| 24 | Lewis et al 1980 | 56 | F | Mandible | Posterior | Hypoesthesia | Radiolucent | Angiolipoma |
| 25 | Steiner et al 1981 | 50 | M | Mandible | Ramus and body | Asymptomatic | Radiolucent | Lipoma (Periosteal) |
| 26 | Polte et al^.^ 1976 | 39 | M | Mandible | Posterior | Hypoesthesia | Radiolucent | Angiolipoma |
| 27 | Johnson 1969 | 21 | M | Mandible | Posterior | Pain & swelling | Radiolucent | Lipoma |
| 28 | Newman 1957 | 65 | M | Mandible | Posterior | Asymptomatic | Radiolucent | Fibrolipoma |
| 29 | Oringer 1948 | 37 | M | Mandible | Posterior | Pain& pressure | Radiolucent | Lipoma |
| 30 | Present case | 39 | M | Mandible | Anterior | Tooth displacement | Radiolucent | Fibrolipoma |

*The case presented with simultaneous diagnosis of Jacob’s disease
